# Parents’ Qualitative Perspectives on Child Asking for Fruit and Vegetables

**DOI:** 10.3390/nu9060575

**Published:** 2017-06-05

**Authors:** Alicia Beltran, Teresia M. O’Connor, Sheryl O. Hughes, Debbe Thompson, Janice Baranowski, Theresa A. Nicklas, Tom Baranowski

**Affiliations:** USDA/ARS Children’s Nutrition Research Center, Department of Pediatrics, Baylor College of Medicine, Houston, TX 77030, USA; abeltran@bcm.edu (A.B.); teresiao@bcm.edu (T.M.O.); shughes@bcm.edu (S.O.H.); dit@bcm.edu (D.T.); jbaranow@bcm.edu (J.B.); tnicklas@bcm.edu (T.A.N.)

**Keywords:** fruit, vegetables, asking skills, parenting style, children

## Abstract

Children can influence the foods available at home, but some ways of approaching a parent may be better than others; and the best way may vary by type of parent. This study explored how parents with different parenting styles would best receive their 10 to 14 years old child asking for fruits and vegetables (FV). An online parenting style questionnaire was completed and follow-up qualitative telephone interviews assessed home food rules, child influence on home food availability, parents’ preferences for being asked for food, and common barriers and reactions to their child’s FV requests. Parents (*n* = 73) with a 10 to 14 years old child were grouped into authoritative, authoritarian, permissive, or uninvolved parenting style categories based on responses to questionnaires, and interviewed. Almost no differences in responses were detected by parenting style or ethnicity. Parents reported their children had a voice in what foods were purchased and available at home and were receptive to their child’s asking for FV. The most important child asking characteristic was politeness, especially among authoritarian parents. Other important factors were asking in person, helping in the grocery store, writing requests on the grocery shopping list, and showing information they saw in the media. The barrier raising the most concern was FV cost, but FV quality and safety outside the home environment were also considerations.

## 1. Introduction

Fruit and vegetable consumption has been inversely correlated with all-cause mortality, especially cardiovascular disease mortality [1], and with hypertension, coronary heart disease, and stroke [2]. Despite these health benefits, children’s FV consumption is below recommended levels [3]. Focusing on children is important because establishing healthy eating behaviors at any child age tracks into adulthood [4]. A determinant of child FV consumption is home availability [5], i.e., having the item in the home (e.g., carrots in the refrigerator) [6]. 

Parents influence their children’s dietary intake [7]. Keeping healthy foods in the home and unhealthy foods out of the home (i.e., home availability) were consistently the most important parental influences on child intake [8]. In addition, parents influence children’s eating behavior through their parenting style [9], i.e., the emotional tone set by the parent for the parent–child relationship. Four general types of parenting style have been identified: authoritarian (highly demanding, and controlling; low emotional warmth), authoritative (highly demanding and controlling; high emotional support and responsiveness), permissive/indulgent (low control and non-demanding, high emotional support and responsiveness), and uninvolved (low demanding, low responsiveness) [10,11]. Parenting style was related to the healthfulness of joint parent–child food shopping selections [12]. Uninvolved parenting style moderated the relationship of child emotional eating and BMIz scores [13]. Maternal indulgent feeding style (i.e., parenting style specific to food) and restrictive parenting practices were related to BMIz score increase over an 18 month interval [14]. Ethnic group differences have been detected in parenting style [15]. 

Parent–child relationships, however, are a two-way interaction [16]. Children have influenced home food availability by expressing their preferences [17,18] and making requests [19]. Children have developed knowledge, skills, and values for decision making to influence purchases for the home [20,21]. One strategy to increase home FV availability is to teach children to effectively ask parents for their favorite FV. Early adolescence (e.g., 10–14 years old) is a time when children are beginning to establish independence [22], and thus an ideal time to learn new ways to relate to parents. A role-playing intervention improved child asking and negotiation skills and showed a positive effect on home FV availability [23], while another reported that a school based intervention increased parent report of child asking for FV [24]. Asking behaviors at baseline among fourth or fifth grade children predicted home FV availability, but small increases in asking behaviors did not increase home FV availability [25]. Children 12–15 years of age expressed reluctance to ask for FV due to the anticipated negative reaction of their parents [26], which may affect how they ask for FV, and depress impact. Furthermore, parents with the alternative parenting styles may respond differently to various ways of child asking. 

Given child reluctance to ask parents to increase FV at home [26], more effective asking interventions may be created if nutrition education interventionists understood how parents might respond to their child’s FV asking behaviors and whether these responses vary by parenting style. Given ethnic group differences in parenting style [15], it would also be valuable to understand differences in parent responses by ethnic group.

This study explored how parents from different ethnic groups with different parenting styles would best receive asking for FV from their 10–14-year-old child, and thereby provide the basis for an intervention teaching children asking skills to enhance home FV availability. 

## 2. Materials and Methods 

### 2.1. Study Design

This study was conducted in two phases in 2006. Phase I was a cross sectional online survey that assessed parenting styles. Parents were categorized by parenting style (i.e., authoritative, authoritarian, permissive, or uninvolved). Phase II was an intensive telephone interview with the parent primarily responsible for home food purchase and preparation. Participants were stratified by parenting style and ethnic group based on survey responses. While the project was based in Houston, TX, USA, and the more intensive recruitment activities were located in Houston, respondents from across the USA could participate since inclusion required only completing a web-based questionnaire and responding to a telephone interview. Participants were asked in the survey to select the state and city in which they were located. 

### 2.2. Sample and Recruitment

Eligible participants were parents or guardians with a 10–14-year-old child, had Internet access, were English or Spanish speaking, and were the person in the household responsible for home food purchase, preparation, and serving. Participants were excluded if their 10–14-year-old child had a health condition that affected their dietary intake. 

Participants were recruited via a national Children’s Nutrition Research Center newsletter, the Children’s Nutrition Research Center volunteer list and webpage, health related electronic mailing lists; flyers, a health fair, and radio advertisements targeted to the African American population. Since African American children tend to eat fewer FV [27] and are less likely to participate in research [28], an attempt was made to increase participation from this group. This convenience approach to recruitment and enrollment lasted nine months. A total of 537 participants entered the website and consented to participate, 8 participants declined to participate; of these, 198 parent/guardian and child pairs (36.3%) completed the online survey. Twenty-two participants did not qualify because they were not the parent or guardian of the child and one participant did not qualify because the child did not live with the parent. We have no data for why the 316 participants who consented electronically did not complete the survey. 

### 2.3. Procedures

The study website informed parents about the purpose of the study, assured confidentiality and obtained online consent to participate for self and child. The parent then completed a screening and demographic questionnaire, the Children’s Report of Parental Behavior Inventory (CRPBI-30) (Schludermann EH & Schludermann SM, 1988, unpublished results) adapted to parents (English or Spanish). After the parent completed the questionnaires, their 10–14-year-old child completed online assent, and answered the CRPBI-30 for children and youth [29]. 

The parent/guardian–child pairs who met the inclusion criteria were then categorized on parenting style calculated from the child CRPBI-30 responses. The goal was to enroll 100 participants: 40 authoritative and 40 authoritarian participants with 10 African American, White, Hispanic, and Other in each of these categories; and 10 participants each in the permissive and uninvolved cells. In our experience with research on parenting, theoretical saturation is usually attained with 10 or fewer participants, thereby providing an adequate sample for ethnicity differences within parenting style groups. Theoretically, we expected the permissive and uninvolved parenting style parents would be less likely to be responsive to child asking behaviors. For example, permissive parents would be expected to allow any food (healthy or not) the child wanted into the home; and uninvolved parents would not be expected to respond to any entreaty. Also, based on previous experience we knew there would be fewer permissive and uninvolved respondents, likely reflecting their indifference to participating in such projects. We continued enrollment for as long as participants were agreeing to be interviewed. The parent was then contacted for the 45-min telephone interview. Interviews were pilot tested. All interviewed parents were compensated $25. The Institutional Review Board of the Baylor College of Medicine approved the protocol and procedures. 

### 2.4. Measures

The demographic and screening questionnaire included questions about ethnicity, parent education and parent household role. The revised children and youth report of parental behavior inventory questionnaire (CRPBI-30) is a short (30 item) version of the 108-item questionnaire (Schludermann EH & Schludermann SM, 1988, unpublished results), which measures three dimensions: acceptance/rejection, psychological control/autonomy, and firm/lax control. Each item was answered on a three-point scale (1 = not like my parent or guardian, 2 = a little like my parent or guardian, and 3 = a lot like my parent or guardian). In a sample of older adolescents reporting on their mothers, Cronbach’s alphas and test–retest correlations were 0.75 and 0.84 for acceptance (10 items), 0.72 and 0.84 for psychological control (10 items), and 0.65 and 0.79 for firm control (10 items), respectively (Schludermann EH & Schludermann SM, 1988, unpublished results) and dimensions were associated with Family Satisfaction as measured by Olson’s Family Satisfaction Scale [29]. 

### 2.5. Qualitative Interview 

A semi-structured interview script was designed by researchers with expertise in child feeding behavior and qualitative interview techniques. The script guided the interview; prompts were used to assess different aspects of the questions while probes were used to expand or clarify the responses (see Table 1). The script explored the family rules about foods eaten at home, participation, and influence of the child in the decision making of the foods available at home, times when parents were willing to talk with the child about foods, ways in which the child could best ask the parent for foods, parent reactions towards specific child FV requests, and barriers to comply with their child’s request. 

### 2.6. Data Analysis

As prescribed by the originators, children’s reports on two of the dimensions: acceptance and firm control were used to categorize their parents on the parenting style category based on median splits from the validation studies (Schludermann EH & Schludermann SM, 1988, unpublished results): authoritarian (high control, low acceptance), authoritative (high control, high acceptance), permissive (low control, high acceptance), and uninvolved (low control, low acceptance). Child responses were used to avoid the possible confounding of common response bias of parent report of parenting style with parent responses to the interview.

Audio-recordings were transcribed verbatim and transcriptions checked against audio-recordings prior to analysis to ensure accuracy. Analysis was conducted in phases: first, separate responses were identified on the transcripts, and entered into Excel (version 12, 2007 Microsoft Office Excel, Microsoft Corp, Redmond, WA, USA); coding was performed manually with thematic codes [30,31] reflecting the questions asked. Within questions, codes were derived as the classification proceeded. Interview response codes were grouped by parenting style and ethnicity to assess possible differences. Given the large number of interviews, coding was conducted independently by six staff members and a coordinator. All transcripts were coded by two coders. The coordinator discussed discrepancies with the independent coders until consensus was established; codings were revised based on the consensus opinion. Comparison summary tables were created to assess differences by ethnicity and then by parenting style. 

## 3. Results

Seventy-three participants (36.9% of the 193 completing the web-based questionnaire) were interviewed: 36 authoritative, 30 authoritarian, 5 permissive, and 2 uninvolved parents (Table 2). The majority were mothers (98.6%) with an average 40.0 ± 5.0 years of age. More parents of girls were interviewed (60.3%). Participants were heavily sourced from Houston (55%), but came from across the US (Table 2). Since no or only slight differences in responses were detected by parenting style, or by ethnicity in the authoritarian and authoritative categories, findings are presented by questions in the script. The few differences by parenting style and ethnicity are noted. 

### 3.1. Influence of the Child on the Food and Beverages Bought for Home

When asking parents how much influence their child had on the foods and beverages purchased for home, most of the parents (88%) said their child had a lot of, or some, influence. Three ways in which the child influenced the parent included: the child decided the type of food; the parent controlled the situation, but allowed the child some input upon request; and the parent asked what the child wanted to eat. “...somewhat my child has a say, because if he’s not going to eat it, it’s no use for me buying it. I have to buy something that I know he’s going to eat, or he wants.” (Interview 380, Authoritarian–African American) 

Some parents (23%) said the child influenced them to buy what the child liked, implying they do not have to ask the child. A few parents (11%) reported the child had a say in food purchases depending on the type of food. If the parent considered the food healthy the parent would buy the requested food, but if the parent considered the food unhealthy, the parent would not purchase it. Some parents (37%) said their child had some say by adding food items to the shopping list, while others (52%) said their child went grocery shopping with them. 

### 3.2. Types of Food Requested by the Child and the Parents’ Reaction 

Children requested a variety of foods; whether parents bought the requested food depended on the type of food. If the food requested was chips or cookies, a small group (11%) would not buy it. Another factor influencing the purchase was budget. A minority (7%) suggested buying a healthier version of the requested food, or buying the food for limited occasions or quantities. Only 14% of parents reported buying the food without any restrictions, in order to please the child. “...if they’re saying, ...can we get green grapes and red grapes, ...if it is something healthy and they ask for it, and granted it is affordable, ...and in the budget ...they can have it, because I encourage them to eat good foods.” (Interview 286, Authoritarian–White) 

### 3.3. Times or Channels for the Parent and Child to Talk about Foods

Most parents (73%) reported that a good time to discuss purchasing a food was in the car, because they had time to talk, and the family was together as a captive audience. However, a small number of parents (18%) reported talking in the car was not a good time. Most parents (89%) reported mealtimes as a good time, preferring dinnertime, because they are together as a family, and talk about the day with food present. Another good time was while making the grocery list (53% of parents). “Usually ...at dinner, or ...because we spend a lot of time in the car driving to different activities and that’s always a very good time for us to talk.” (Interview 650, Authoritative–Other Ethnicity) 

Some parents (30%) reported anytime was good to talk about food. The grocery store (18%), while cooking (10%), and before or after school (15%) were other times mentioned by small numbers of parents. 

### 3.4. How Parents Liked Their Child to Ask for Food

Some parents (44%) reported a good way was just asking them in person. About 40% thought their child should ask in a polite manner. This was mentioned more often by the authoritarian parents (18/30) than authoritative (7/36) or permissive (2/5) parents. Across all groups, some of the parents (37%) indicated a good way was asking or selecting the food at the grocery store. “... if they …asked me, “Mom, could you get me something today?” and they said it in a nice tone with a good attitude and they were happy about it”. (Interview 722, Uninvolved–White)

Another good way was writing the food on the grocery list (52% of parents). “I keep a list that they can add to. But they pretty much know, ...what’s okay to put on there.” (Interview 310, Authoritative–White) Some parents (26%) reported another way to ask was showing them information from the TV or child magazine ads. 

When asked whether a child could ask too often, a small group (8% of parents) reported they would prefer not having the child ask too often (not further specified). However, other parents (32%) indicated they would get what the child asked for, depending on the type of food and the attitude of the child.

### 3.5. Common Barriers to Comply with Child Requests

Most parents (84%) said they would buy FV if their child asked; further, they reported that buying only the FV their child ate was not a barrier. However, a small number (15%) indicated they would be careful with the amount of FV purchased, if no one else at home ate that particular FV. “…if one liked it and the rest did not, I would just buy less of it, but I would still buy it.” (Interview 311, Permissive-White) 

Across all groups, some parents (58%) reported being willing to buy the FV even if it had to be thrown away in the past. However, if this was the case they would purchase less of it or less frequently in the future and a few authoritarian parents would remind the child this would be the last time. A small number of parents (26%) reported they were not likely to purchase the FV if it had been thrown away in the past. 

Parents’ FV preferences were not a barrier to buying FV for their child. Some parents (42% would buy an expensive FV if the child asked. Others (52%) would buy the requested FV depending on their budget and only if the FV were in season. A small number of parents (19%) would limit the quantity purchased. “If it’s expensive but still a little reasonable I will buy it as long as it’s something healthy and I know they will eat it...” (Interview 436, Authoritative–Hispanic) 

Time for FV preparation and lack of knowledge about preparation were not considered barriers for most parents (66%). Some parents (19%) would purchase the FV and prepare them only when they had time to do so. Only a few parents (16%) reported the lack of time and knowledge to prepare FV to be a barrier and therefore not likely to buy them. 

### 3.6. Reactions to Specific FV Requested by Their Child

Most parents (78%) had a positive reaction toward buying 100% fruit juice for breakfast, and F and V and dip for a snack for their child. A few parents (11%) reported not likely buying 100% fruit juice because of the high sugar content and price. A few parents (10%) would not buy V and dip for a snack because they believed their child would not eat it or was not in their budget. Most parents (59%) already bought salad for home, while some parents (11%) would not buy salad for home because of the quality of salad and beliefs their child would not eat it. “Maybe a fruit salad but a vegetable leaf salad, she will not touch it with a ten-foot pole.” (Interview 439, Authoritarian–Hispanic) 

Many (84%) reported they would make their child’s favorite V for dinner when available. Most parents (75%) would add a F or V to the grocery list, if asked, and buy F at a restaurant (59%), even though a few (5%) thought it was expensive. However, some parents (36%) were not likely to buy F at a restaurant because of perceived low quality, high price, and preference to eat F at home. “No, I don’t buy fruit or vegetables ...you don’t know what they put inside, ...what kind of seasoning. ...how they prepare it.” (Interview 146, Authoritative–African American)

Most, but not all, parents (79%) said they would very likely buy salad or a V at a restaurant for their child. 

## 4. Discussion

Availability of FV at home has consistently been shown to be related to child vegetable intake [32], but reticence in child asking to increase FV has been reported [26]. This study explored how parents would best respond to their 10–14-year-old child asking for FV. In general, parents appeared to be receptive, but the receptiveness of some appeared to be tempered by whether the food requested was considered healthy, and the parent retained control over the situation by limiting the quantity purchased. Although differences in parental acceptance of child asking was expected by parenting style and ethnicity, only slight differences were found in parent responses across authoritative and authoritarian styles and ethnicities. Thus, tailoring an intervention to parenting style or ethnicity does not appear to be needed. Alternatively, an intervention should take steps to appeal to all ethnicities or to be ethnic neutral [33].

Most of this literature has emphasized how parents do or can influence children, and so few references exist for comparison on how children can influence parents and the home food environment [26]. Children directly and indirectly had an influence on what foods were available at home. This is the first report of parent receptiveness to child asking. Parents expressed some control over the food available at home, but kept in mind the child’s preferences when buying food, or allowing the child to select the food. Similar observations have been reported about how child preferences influence food availability at home [17]. Furthermore, children’s influence on food purchases has been fully recognized and successfully used by marketers [19,20]. A community-based gardening intervention showed children influenced the families’ decision making [34]. Marketers have identified influence tactics used by the children to persuade their parents to fulfill their needs e.g., consultation, where the child involves a parent in making a decision [19] or ingratiation where the child gets the parent in a good mood, before asking the parent to comply with their request. Thus, teaching a child to state their FV preferences and ask for FV appear to be promising techniques for helping increase availability of FV in the home and thereby its consumption. Future research should investigate these four pathways of child influence in the context of FV asking. 

Parents were aware of the importance of having “healthy food” available at home, and limiting availability of unhealthy food. Concern of parents to have healthy food available in the home has been shown in other studies [35]. Thus, since FV are generally considered healthy, most parents would appear to be receptive to FV requests.

The most important child asking characteristic was politeness, especially among authoritarian parents. Another important factor was the channel for asking, whether in person or by helping in the grocery store, writing it on the grocery list or showing parents the information in the media. These factors have been effective at influencing parents’ purchases in other settings [19,20]. Any intervention encouraging stating preference or asking for FV should emphasize politeness on the child’s part in each of the channels of asking irrespective of parenting style.

Although some parents reported situational barriers, most offered to still purchase FV requested, but in limited quantities or at different times. The barriers that raised more concern were FV costs and the quality and safety of the FV obtained at restaurants. The intervention might teach children to have realistic expectations for parent responses to their asking. Alternatively, this could be an opportunity to combine math training, microeconomics, and nutrition by teaching somewhat older children relative pricing, amounts purchased, and household budgeting.

A limitation of this study was our inability to recruit substantial numbers of parents who practiced permissive and uninvolved parenting, which restricts the comparisons and the generalizability of the findings. Only 36.3% of mothers who completed the online consent also completed the online survey. Mothers lived in diverse locations across the US, and we do not know their reason(s) for participating, or others for not participating, which may bias the results. Thus, the sample may not be representative of all mothers of children this age, thereby limiting generalizability. Since family structure influences food choices in households [36], further research is needed on how the composition of the household affects family negotiations towards foods available at home. 

## 5. Conclusions

Parent–child communication is bidirectional. Parents showed openness to complying with their child FV requests, as long as certain minimal criteria were met. Children need to learn to ask parents for FV in a polite manner. The training should specifically address alternative channels, e.g., during meals, while preparing the grocery list, in the kitchen when the parent is cooking, in the car, or at the grocery store. Strategies should improve the asking skills to overcome some of the parents’ perceived barriers like the lack of time to prepare FV, cost of FV, and FV food safety out of the home environment. Tailoring an intervention to parenting style or ethnicity does not appear to be necessary.

## Figures and Tables

**Table 1:** Questions used to guide the semi-structured interview

1. How much say, if any, does your child have about the foods and beverages you buy for home?
2. If your child wanted to talk with you about the foods they like or don’t like, when are the best times or situations for them to talk with you?
3. If your child wanted to have specific foods available at home, describe the best way for them to ask you.
a. What other ways, other than asking, could they use to let you know about specific foods they wanted at home?
4. How likely would you be to buy fruit or vegetables if your child asked for them?
a. How would your response to your child change:
• If no one else in your home would eat the fruit or vegetable?
• If you’ve bought this fruit or vegetable in the past but had to throw it away?
• If you personally do not like this fruit or vegetable?
• If the fruit or vegetable is expensive?
• If the fruit or vegetable cannot be prepared quickly?
• If you don’t know how to prepare this fruit or vegetable?
5. How likely would you be to do the following, if your child asked you to:
• Buy 100% fruit juice for breakfast?
• Buy fruit for an after-school snack?
• Buy vegetables and dip for a snack (e.g., carrots and low fat ranch dip)?
• Buy a salad for home?
• Make a salad for home?
• Make their favorite vegetable for dinner?
• Add a fruit or vegetable to the grocery list?
• Buy fruit at a restaurant?
• Buy a salad at a restaurant?
• Buy a vegetable at a restaurant?

**Table 2:** Parent–child demographic characteristics.

Characteristic	*n* (%)	M (SD)
Total parent–child interviews	73 (100.0)	
Age of 10–14 yo child (years)		
10	13 (17.8)	
11	13 (17.8)	
12	17 (23.3)	
13	17 (23.3)	
14	13 (17.8)	
Parent Age		39.97 (5.89)
Child gender		
Male	29 (39.7)	
Female	44 (60.3)	
Parent gender		
Male	1 (1.4)	
Female	72 (98.6)	
Child Race/Ethnicity		
White	22 (30.1)	
AA	19 (26.0)	
Hispanic	23 (31.5)	
Other	9 (12.3)	
Parent Race/Ethnicity		
White	27 (37)	
AA	20 (27.4)	
Hispanic	20 (27.4)	
Other	6 (8.2)	
Parenting style		
Authoritative	36 (49.3)	
Authoritarian	30 (41.1)	
Permissive	5 (6.8)	
Uninvolved	2 (2.7)	
Highest Parent Education		
HS Graduate or less	10 (13.7)	
Some college/technical school	22 (30.1)	
College graduate	23 (31.5)	
Post graduate study	18 (24.7)	
Highest Household Education		
HS Graduate or less	11 (15.1)	
Some college/technical school	21 (28.8)	
College graduate	19 (26.0)	
Post graduate study	22 (30.1)	
State of participants		
California	2 (2.7)	
Colorado	1 (1.4)	
Maine	1 (1.4)	
Minnesota	2 (2.7)	
North Carolina	1 (1.4)	
New Mexico	4 (5.4)	
Oklahoma	1 (1.4)	
Oregon	1 (1.4)	
Texas Houston	40 (55)	
Other Cities	18 (24.6)	
Tennessee	1 (1.4)	
Wyoming	1 (1.4)	

Percentages that do not sum to 100% are due to rounding. Legend: AA = African-American; HS = High School.
